# Direct Targeted Degradation of Transposon RNAs by the Non-Canonical RNAi Pathway of the Fungus *Mucor lusitanicus*

**DOI:** 10.3390/ijms26062738

**Published:** 2025-03-18

**Authors:** Ghizlane Tahiri, Carlos Lax, Francisco E. Nicolás, Victoriano Garre, Eusebio Navarro

**Affiliations:** Departamento de Genética y Microbiología, Facultad de Biología, Universidad de Murcia, Campus de Espinardo, 30100 Murcia, Spain; ghizlane.tahiri@um.es (G.T.); carlos.lax@um.es (C.L.); fnicolas@um.es (F.E.N.)

**Keywords:** NCRIP, sRNAs, TEs, regulation, genome stability, GremLINE1

## Abstract

*Mucor lusitanicus* has emerged as a model organism for studying RNAi in early-diverging fungi. This fungus exhibits intricate RNAi pathways that play crucial roles in regulating gene expression, destroying invasive exogenous genetic material, and controlling the movement of transposable elements (TEs) to ensure genome stability. One of the most fascinating RNAi pathways of this fungus is the non-canonical RNAi pathway (NCRIP), which is independent of Dicer and Argonaute proteins and uses the atypical RNase III R3B2 to degrade specific target messenger RNAs (mRNAs), playing an essential role in genome stability and virulence. Despite accumulating data suggesting that this pathway is a degradation mechanism, there has been no conclusive evidence. Here, we conducted a comparative transcriptomic analysis of mRNA and small RNAs regulated by *r3b2*, identifying 35 direct NCRIP targets. Most of these direct NCRIP targets correspond to TEs, highlighting the significant role of this RNAi pathway in TE control. Detailed functional analysis of the NCRIP targets confirmed the crucial role of *r3b2* in regulating gene expression of protein-coding genes and controlling TEs other than centromeric GremLINE1 transposons, emphasizing the important role of *r3b2* in genome stability. Interestingly, the RNAs of the NCRIP targets harbor a unique motif consisting of CAG repeats which are known to form hairpin structures which are targeted by RNA interference. Additionally, the generation of transformants expressing mRNAs containing the luciferase reporter gene along direct NCRIP targets reveals that this RNAi pathway is a true degradation mechanism for specific mRNAs. These results are expected to contribute to the understanding of the regulation of the NCRIP pathway through the analysis of its direct targets identified here.

## 1. Introduction

Members of the order Mucorales are the causative agents of the human infection mucormycosis [1], a rare disease that is rising globally and has a high mortality rate especially in disseminated infections [2]. These filamentous fungi affect mainly immunocompromised patients with impaired immune systems, such as neutropenia, hematological malignancies, and organ transplantation [1]. Additionally, new risk factors, such as renal failure and post-tuberculosis, have emerged recently [1]. Nevertheless, cases of apparently healthy people who suffered from burns, trauma, or surgeries have been reported [2,3]. In western countries, hematological malignancies and organ transplantation are the most common risk factors, whereas in Asian countries, diabetes mellitus is the most prevalent underlying condition [2]. The infection process starts with the inhalation of spores into the lungs, consumption of contaminated food or through wounds, or disrupted skin [4]. In patients with reduced phagocytic activity, including those with diabetic ketoacidosis, the Mucorales proliferate or persist, leading to serious consequences. Iron uptake also plays a crucial role in mucormycosis because patients with high levels of iron in the blood are more susceptible to suffering from mucormycosis [4]. Over the last ten years, an increase in the incidence of mucormycosis has been registered, probably due to the rise in immunocompromised individuals [5,6]. Most recently, a huge increase in numbers of cases of mucormycosis in patients suffering from Coronavirus disease 2019 (COVID-19) caused by severe acute respiratory syndrome coronavirus 2 (SARS-CoV-2) has been reported [7]. Although COVID-19 itself may affect the immune system resulting in vulnerability of the patients to mucormycosis, poor diabetic control and the administration of systemic corticosteroids has greatly contributed to the occurrence of the disease in severe and critical COVID-19 patients [6]. The current situation has led the World Health Organization (WHO) to include this group of fungi within the high group of importance in its list of first fungal priority pathogens [8].

*Mucor lusitanicus* (formerly named *M. circinelloides* f. *lusitanicus*) [9] belongs to the Mucorales group and has been extensively used as a model organism for the study of mucormycosis [10]. Two main RNA interference (RNAi) mechanisms have been described in this fungus: the canonical pathway and the non-canonical RNAi pathway (NCRIP) [11], also called the alternative [12]. The first one operates in a classical way, where dsRNA is cleaved by the Dicer enzymes into small interfering RNAs (siRNAs) of 20-25-nt in length. These double-stranded RNA (dsRNAs) fragments are loaded in an RNA-induced silencing complex (RISC), whose main component is Argonaute, where a strand is degraded, while the other is used to direct the complex to the complementary sequences [13]. In addition of protecting the genome from invading nucleic acids, this mechanism also controls gene expression by the production of endogenous short RNAs (esRNAs) derived from protein-coding genes, known as exonic-siRNAs (ex-siRNAs) [14]. Several studies reveal this pathway is involved in the control of physiological and developmental processes, such as vegetative growth, sporulation and accelerated autolysis in response to nutrient starvation [13,15].

The NCRIP is characterized by the central role of the atypical RNase III R3B2 and the absence of Dicer and Argonaute components. This RNase is unique among the RNases III because although it can bind both single-stranded RNAs (ssRNAs) and dsRNAs, it cleaves only ssRNAs [16]. In addition, it is conserved only among Mucorales, and its RNase III-like domain shows a limited similarity with the RNase III domain of several bacteria of the order Burkholderiales, suggesting that it could be originated from a horizontal transfer between *Burkholderia* and an ancestor of the order Mucorales [11]. In addition to R3B2, three RNA dependent RNA polymerases (RdRP) participate in the NCRIP. Their functions are not fully understood, although it is suggested that they bind to target transcripts and make a short complementary strand that mark them for degradation by R3B2, producing esRNAs that are sense to the mRNA, contrary to the canonical pathway that produces sense and antisense small RNAs (sRNAs) [17]. These esRNAs are additionally characterized by their random size distribution in comparison with those generated by the canonical RNAi pathway and are enriched in uracil in the penultimate position. These esRNAs are known as *rdrp*-dependent degraded RNAs (rdRNAs) and include most ex-siRNAs derived from the canonical pathway, specifically Class III [11].

Interestingly, recent studies have shown the implication of the NCRIP in the virulence of *M. lusitanicus*, since mutants lacking the main proteins involved in this RNAi pathway, RdRP1 and R3B2, exhibit a reduced virulence when assayed in mouse models. These mutants show a constitutive activation of the response to phagocytosis, indicating that NCRIP represses its target genes during saprophytic growth and activates its transcription during stressful conditions, such as during the phagocytosis of spores [18].

An intriguing additional canonical RNAi pathway has been described in *M. lusitanicus*, known as the epigenetic pathway, because it does not require any genomic change to promote resistance to antifungal drugs [19,20]. In response to exposure to the drug or prodrug, this mechanism produces siRNAs complementary to mRNAs of the target protein or proteins that metabolized the prodrug, resulting in drug-resistant epimutants that show low levels of the corresponding mRNAs. This resistance is transient because they restore the wild-type phenotype when grown in free-drug media. Interestingly, this epigenetic RNAi-dependent pathway is repressed by the NCRIP because mutants in the latter enhance the production of drug-resistant epimutants [17,19]. In addition, the NCRIP also modulates the activity of the canonical RNAi pathway in controlling the movement of the centromeric retrotransposon Grem-LINE1 to maintain genome stability [12,18].

Despite the importance of the NCRIP in gene regulation in *M. lusitanicus* and probably other mucoralean fungi, no work has performed a comprehensive analysis to identify the direct targets of this RNAi pathway. Previous works have characterized the sRNAs produced and/or mRNA regulated by the NCRIP [11,12,18], but they have not actively searched for direct targets of this RNAi pathway. In addition, the mechanism underlying the degradation of specific mRNA by the NCRIP remains largely unexplored. In this work, sRNAs and mRNAs from the *r3b2*Δ mutant and a wild-type strain were sequenced in parallel and compared to identify the genes directly regulated by the NCRIP. In addition, analysis of the mRNA degradation using the luciferase gene as a reporter confirmed that the NCRIP regulates gene expression through targeted degradation of specific transcripts.

## 2. Results

### 2.1. Genes Regulated by NCRIP Under Non-Stressful Conditions

**Figure 1:**
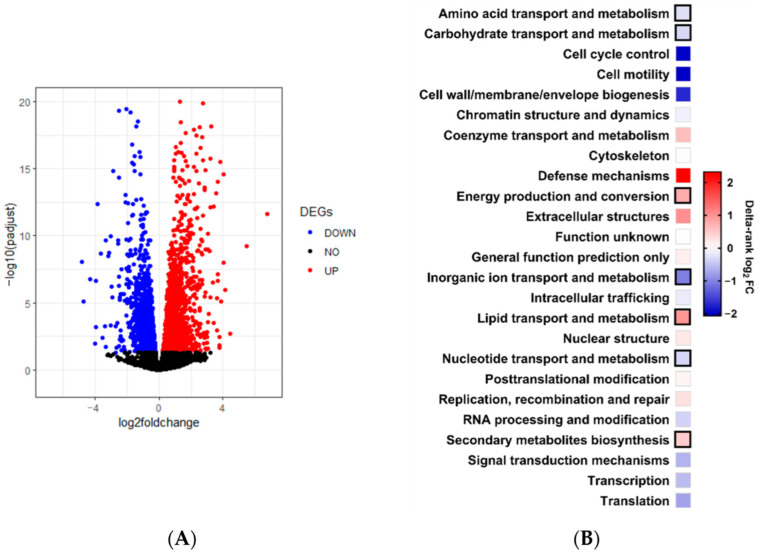
Genes regulated by *r3b2*. (**A**) Volcano-plot showing DEGs between *r3b2*Δ mutant and wild-type strain. Statistically significant (*P*adj < 0.05) downregulated and upregulated genes in the mutant are shown in blue and red, respectively. Black dots correspond to non-significant DEGs (*P*adj ≥ 0.05). (**B**) Enrichment analysis by the KOG classification of the 989 DEGs showing absolute log_2_FC ≥ 1. Black squares indicate significant enrichments (Fisher’s exact test, *p* ≤ 0.05) in the mutant strain compared with the wild-type strain.

To identify the primary targets of the NCRIP, we performed a transcriptomic analysis of the gene expression profiles obtained from high-throughput sequencing (RNA-seq) of both mRNAs and sRNAs from the mycelium of the wild-type and the mutant in *r3b2* (*r3b2*Δ) strain, lacking the NCRIP activity, grown in non-stressful conditions on solid YPG media pH 4.5 for 24 h. The analysis of mRNA from three biological replicates of both strains was conducted to identify the differentially expressed genes (DGEs). Principal component analysis (PCA) revealed that biological replicates from the mutant and wild-type strains clustered together (Supplementary Figure S1), indicating that one component, a deletion of *r3b2*, explained practically all the differences detected between the mutant and the wild-type replicates. As a result of the transcriptomic analysis, we found 3247 DEGs (Figure 1A; Dataset S1) under these non-stressful conditions between the *r3b2*Δ and the wild-type strains. To increase the stringency of our analysis, we focused on the 989 genes that exhibited an absolute Log_2_FC ≥ 1. Of these DEGs, approximately two-thirds (647) were upregulated, while the remaining third (342) were downregulated in the mutant compared to the wild-type strain.

**Figure 2:**
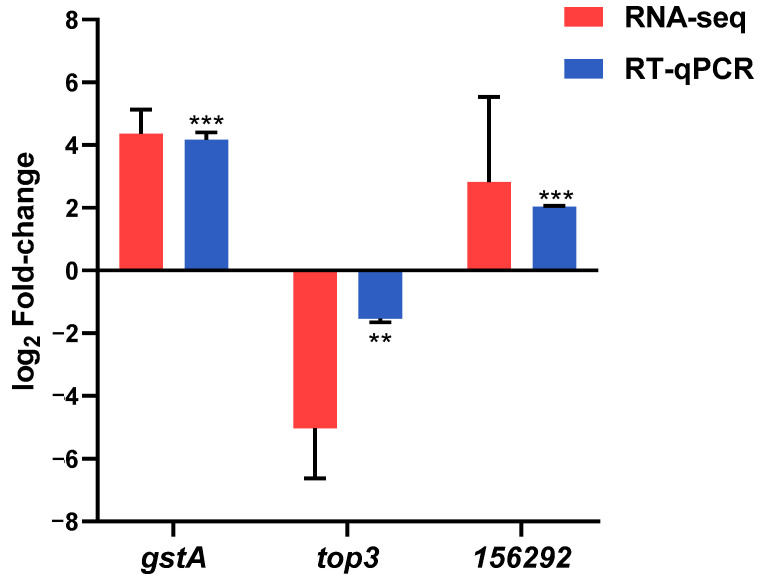
Validation of the transcriptomic results. Relative mRNA levels (mean log_2_FC ± SD) of the three indicated genes determined by RT-qPCR (blue bars) and RNA-seq (red bars) in the *r3b2*Δ mutant in comparison to the wild-type strain. Normalization in RT-qPCR was performed using the elongation factor (*ecf-1*) as a reference. The asterisks indicate significant differences in an unpaired *t*-test: ** for *p*-value ≤ 0.01 and *** for *p*-value ≤ 0.001. The three genes show *P*adj values below 0.004 in the bioinformatic analysis of the RNA-seq data.

Validation of the transcriptomic analysis was carried out by measuring mRNA accumulation of three DEGs by reverse transcription quantitative PCR (RT-qPCR). The selected genes were chosen based on their biological relevance as indirect targets of the NCRIP pathway and their functional diversity. We chose three upregulated genes representing different functional categories, one corresponding to an expressed fragment of a putative transposable element (TE) (ID:156292). The second indirect target (ID: 152804) encoded a putative transporter of the major facilitator superfamily (MFS), named *top3* due to its similarity with the gene in *Saccharomyces cerevisiae*. The third indirect target (ID: 114483) encoded a glutathione-S transferase, named *gstA*. As multiple copies of the last gene sharing high similarity are present in the *M. lusitanicus* genome, primers were designed to align with specific sequences of the identified target. Although the degree of change was different, the RT-qPCR results showed the same trend in expression changes for the three analyzed genes as observed in the RNA-Seq data (Figure 2), supporting the RNA-Seq analysis.

To identify cellular processes possibly controlled by *r3b2* under these saprophytic conditions, we focused only on the 989 DEGs showing an absolute Log_2_FC ≥ 1. The enrichment analysis of eukaryotic orthologous groups (KOG) showed enrichment in processes related to amino acid transport and metabolism, carbohydrate transport and metabolism, energy production and conversion, inorganic ion transport and metabolism, lipid transport and metabolism, nucleotide transport and metabolism, and secondary metabolite biosynthesis (Figure 1B). All those functions reflect that during exponential growth, *r3b2* regulates mainly metabolic processes. Moreover, a detailed analysis of gene function confirmed that even in saprophytic conditions and in rich medium, the *r3b2*Δ mutant showed a stressful profile of gene expression, which was previously demonstrated by the high tolerance to oxidative stress shown by the NCRIP mutants [11,18].

### 2.2. Genes Regulated by the r3b2 Independently on Culture Conditions

**Figure 3:**
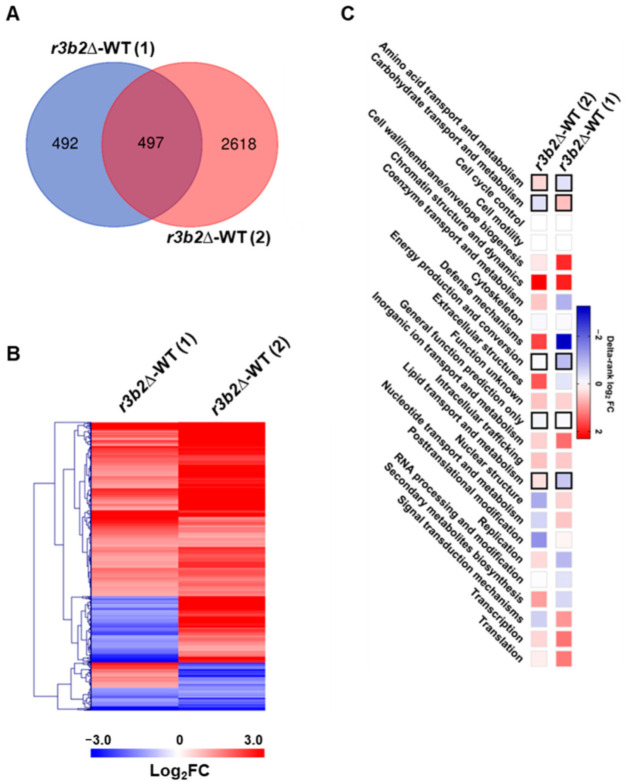
Genes regulated by *r3b2* in two different growth conditions. (**A**) Venn diagram showing the DEGs between the *r3b2*Δ and wild-type strains in samples grown for either 24 h in YPG solid medium (1) or for 5 h in liquid L15 medium (2). (**B**) The heatmap represents the log_2_FC of the common DEGs identified in the transcriptomic analyses 1 and 2. Genes with reduced expression in the mutant relative to the wild-type strain are indicated in red, while genes with induced expression are indicated in blue. (**C**) KOG class enrichment analysis of the common DEGs in the two transcriptomic studies. Black squares indicate significant enrichment processes (Fisher’s exact test, *p* ≤ 0.05) in the mutant. Up-enrichment (red) or down-enrichment (blue) of each KOG class is depicted as a scale of delta-rank values.

While this work identified the genes regulated by *r3b2* when the fungus grows in exponential phase on solid medium and under non-stressful conditions, a previous study determined the genes controlled by this protein in entirely different conditions, as it was grown for 5 h in liquid L15 medium [18]. With the aim of identifying the genes that are regulated by *r3b2* regardless of growth conditions, we compared the DEGs of both studies. This analysis revealed that 497 genes were differentially expressed under the two considered culture conditions and could correspond to core genes regulated by *r3b2* and likely NCRIP (Figure 3A). Most of these common genes exhibited consistent expression patterns across both experimental conditions. However, a small subset displayed contrasting expression profiles: upregulated in one growth condition and downregulated in the other, and vice versa (Figure 3B). The cellular processes controlled by the common genes were largely similar to those observed during exponential growth under non-stressful conditions (Figure 1B and Figure 3C).

### 2.3. Identification of NCRIP Direct Targets

**Figure 4:**
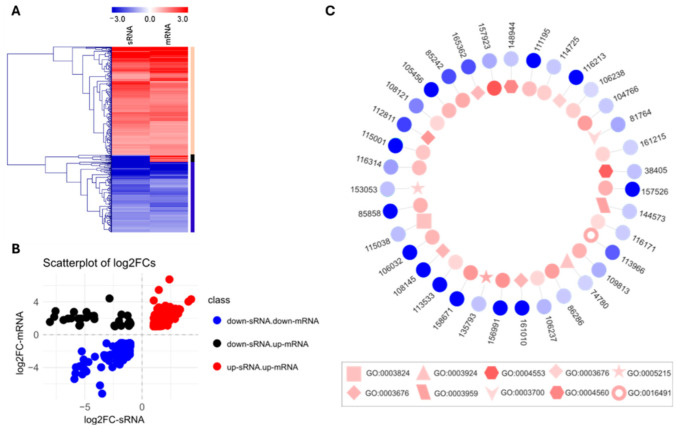
NCRIP targets. (**A**) Comparison of genes showing differential accumulation of sRNAs and mRNAs in *r3b2*Δ mutant in comparison to the wild-type strain. The black bar on the right refers to the direct targets of NCRIP. (**B**) Shows the scatterplot with the log_2_FC values of sRNAs on the *x*-axis and mRNA on the *y*-axis. Each point on the plot represents a gene, with its position indicating the relative change in expression for both sRNA and mRNA. Three distinct clusters can be observed: the red cluster includes genes where both sRNA and mRNA levels are increased. The blue cluster consists of genes with both sRNA and mRNA decreased, and the black cluster contains genes with decreased sRNA and increased mRNA levels, which include the NCRIP direct targets. (**C**) GO terms associated with the direct targets of the NCRIP. The internal layer of the plot represents the expression levels of these genes at the mRNA level, while the external layer shows the expression at the sRNA level. In the external layer, a more intense blue color indicates a lower abundance of sRNAs in the *r3b2*Δ mutant compared to WT. Conversely, in the internal layer, a more intense red color represents a higher abundance of mRNAs in the *r3b2*Δ mutant relative to WT. Circular shapes within the internal layer indicate genes with unknown molecular functions, whereas other shapes correspond to genes with identified molecular functions (see text).

The NCRIP is supposed to regulate gene expression in a hierarchical manner by degrading primary or direct mRNA targets that results in an enrichment of the corresponding sRNAs sense to the transcripts [11]. To identify the direct targets, we selected genes that showed a differential accumulation of sRNAs sense to the transcript and compared this dataset with the DEGs dataset obtained in the deep sequencing of mRNAs (Figure 4). Transcriptomic analysis of the sRNAs mapping genes accumulated by the wild-type strain and the *r3b2*Δ mutant identified 1598 genes that showed differential accumulation of sRNAs (Dataset S2). Comparison of this group of genes with the DEGs revealed that 447 genes were present in both datasets (Figure 4A,B), whereas 542 genes were differentially expressed only at the mRNA level and 1151 genes only showed differences in sRNA accumulation. According to the patterns of mRNA and sRNA accumulation, we established three groups of genes that showed sRNA and mRNA changes in the *r3b2*Δ mutant compared to the wild-type strain (Table 1, Figure 4A,B). Most genes (239) exhibited elevated mRNA and sRNA levels (+sRNAs, +mRNA), followed by the group of genes that displayed (173) reduced mRNA and sRNA levels (−sRNAs, −mRNA). A small group of 35 genes showed the expected pattern for direct target in which the increase in mRNA levels was associated with a reduction in sRNA levels (−sRNAs, +mRNA). Lastly, no gene exhibited an increase in sRNA level and a decline in mRNA degradation (Table 1).

To further understand the functional roles of the RNA interference (RNAi) pathway, a Gene Ontology (GO) analysis was performed with the 35 target genes, specifically focusing on the molecular function (Figure 4C) and biological process (Supplementary Figure S2) categories. The most prominent terms included “nucleic acid binding” (GO:0003676), “oxidoreductase activity” (GO:0016491), “DNA-binding transcription factor activity” (GO:0003700), “catalytic activity” (GO:0003824), “GTPase activity” (GO:0003924), “NADPH dehydrogenase activity” (GO:0003959), “ hydrolase activity, hydrolyzing O-glycosyl compounds” (GO:0004553), “alpha-L-fucosidase activity”( GO:0004560), and “transporter activity” (GO:0005215), indicating that the RNAi pathway predominantly targets genes involved in describing key functions, e.g., “regulating enzymatic processes,” “binding to nucleic acids,” “modulating catalytic activities”. These terms suggest that the NCRIP predominantly targets genes involved in regulating a variety of biological processes, including transcription, metabolic process and transport of molecules. This highlights the diverse roles of RNAi in cellular processes, impacting key regulatory and catalytic pathways.

In addition to the GO enrichment analysis, we performed a domain search with these same targets using InterPro and KOG on the target proteins. This analysis aimed to identify conserved domains and functional sites within the proteins targeted by the RNAi pathway, providing further insights into their potential roles and mechanisms of action. We found that 9 of the 35 targets have domains found in TEs, while the rest included genes encoded an RNA-binding protein, proteins involved in oxidative stress, and microtubule-associated proteins (Dataset S3). Thus, gene ID:144573 encodes a putative oxidoreductase of the Old Yellow Enzyme (OYE) family which includes Oye2/3 from budding yeasts, which are flavin-dependent NADPH oxidoreductases that have roles in oxidative stress and programmed cell death in yeast [21]. Gene ID:113966 encodes a protein that shows similarity with the AIFM2 protein of several species, including human, a flavoprotein oxidoreductase that binds single-stranded DNA, and is involved in the regulation of cellular response to oxidative stress and suppressing ferroptosis [22]. Gene ID:85858 contains a TE similar to ag-JOCK-1; 116213 encodes a C2H2-type zinc finger transcription factor; gene ID:38405 is associated with the glycosyl hydrolase family 3 (GH3) and encodes enzymes like beta-glucosidases, which are involved in carbohydrate metabolism. The presence of the fibronectin type III-like domain suggests potential roles in protein–protein interactions. Gene ID: 74780 is involved in small GTPase activities, specifically within the Ras superfamily, which regulates processes such as cell growth, signal transduction, and vesicle trafficking. The Ras and Rab domains indicate a role in intracellular signaling pathways. Gene ID: 115038 encodes a protein with an AMP-binding enzyme domain, which is involved in the activation of substrates through ATP hydrolysis. The gene is linked to fatty acid metabolism, with a specific role in the ligation of long-chain fatty acids to CoA. Gene ID:135793 encodes a protein in the major facilitator superfamily (MFS), which functions in transmembrane transport. Gene ID:148944 encodes alpha-L-fucosidase, an enzyme involved in the hydrolysis of fucosylated glycoconjugates. Gene ID:153053 encodes a sugar transporter protein from the major facilitator superfamily (MFS), likely involved in the transport of sugars across cell membranes. Gene ID:157923 encodes a serine/threonine-rich galactomannoprotein, involved in cell wall structure and integrity. The protein may play a role in cell adhesion, structural support, and interaction with other cells or the extracellular matrix. Gene ID:116171 encodes a protein related to nucleolar GTPase/ATPase p130 (KOG2992). Gene ID:81764 encodes a protein related to the homeobox family, particularly the HHEX homeodomain protein. Homeobox proteins are transcription factors crucial for regulating development, controlling gene expression during embryogenesis, and maintaining tissue differentiation. Gene ID:108145 encodes a DNA-binding centromere protein B (CENP-B), a protein involved in centromere function. CENP-B helps in the assembly of centromeric chromatin, which is essential for proper chromosome segregation during cell division. Gene ID:85242 is associated with the OTU domain. Eukaryotic proteins containing an OTU domain could mediate proteolytic events involved in signaling associated with the modification of chromatin structure and the control of cell proliferation. Gene ID:86286 encodes a protein with domains related to ice-binding-like adhesive function [23], likely involved in cell adhesion or interactions with the extracellular environment. The “choice-of-anchor A” domain suggests a role in anchoring proteins to cell membranes. Genes ID:104766 and ID:114725 contain a Kelch motif, indicating that they may play a role in protein–protein interactions. Gene ID:109813 encodes a protein associated with the arrestin domain, known for its role in regulating G-protein coupled receptors (GPCRs), particularly in signal transduction. Gene ID:158671 encodes a protein related to the DNase I-like family, which is involved in DNA degradation. Genes ID:165362 and ID:112811 are associated with His-Me finger endonucleases, involved in DNA repair and recombination. Gene ID:161215 is associated with protein interacting with poly(A)-binding protein (KOG2375). Gene ID:106237 is associated with microtubule-associated proteins (KOG3592). Microtubule-associated proteins (MAPs) regulate the dynamics and stability of microtubules, which are critical for cell structure, intracellular transport, and mitosis. Genes ID:116314, ID:113533, and ID:108121 have no specific domains.

### 2.4. Conserved RNA Motifs in the Direct Targets of the NCRIP

**Figure 5:**
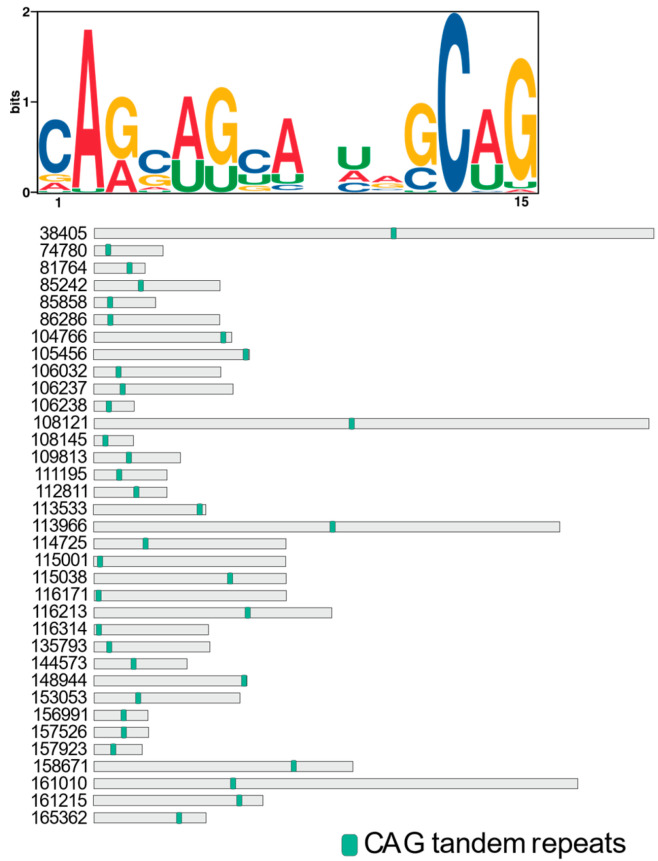
Distribution of the common motif containing the CAG tandem repeats across the RNA sequences of the direct targets of R3B2. Each panel represents a different RNA sequence, with the CAG motif highlighted in green.

The identification of common motifs in the RNA sequences of the direct targets can shed light on the mechanisms of RNA recognition, cleavage and regulation. In this line, we found a motif across the RNA sequences of R3B2 direct targets containing CAG repeats similar to those that are found in specific genes that can cause human genetic disorders such as Huntington’s disease and the spinobulbar muscular atrophy [24]. This motif was consistently found across all target sequences (Figure 5), suggesting a common structural or functional element in the RNA molecules recognized by R3B2. As a control, we performed the same motif search analysis on a set of 35 randomly selected genes to evaluate the specificity of the identified CAG tandem repeats in R3B2 direct targets. In contrast to the R3B2 targets, the CAG motif was not detected in the sequences of random genes. This suggests that the presence of CAG tandem repeats is specific to the direct targets of R3B2 and not a common feature among random gene sequences in the fungal genome. This control analysis supports the notion that the CAG motifs are selectively enriched in the RNA sequences targeted by R3B2, reinforcing their potential role in RNA recognition and regulatory functions within the RNAi pathway.

### 2.5. Analysis of TEs Controlled Directly by the NCRIP

**Figure 6:**
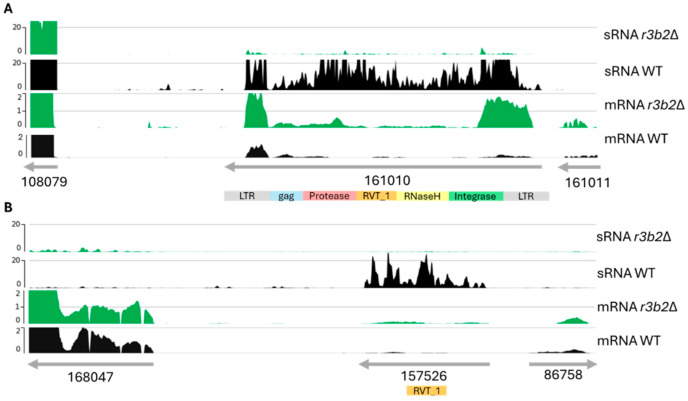
Annotation of transposable elements directly targeted by the NCRIP. (**A**) Structure of putative LTR transposon showing its key structural domains, including the possible long terminal repeats (LTRs), and the conserved protein domains such as protease, reverse transcriptase (RVT_1), RNase H, and integrase. Green and black plots show the coverage of sRNA and mRNA reads mapped to the transposon and their adjacent genes in the WT and mutant (*r3b2*Δ) samples, respectively. (**B**) Structure and expression of a non-LTR transposon. In both cases, the same data from the neighboring genes are shown.

Among the direct targets of the NCRIP in the analyzed conditions, we found nine putative TEs, which were further studied and annotated using the RNAseq data. To further characterize and annotate these TEs, we integrated data from the sRNA and mRNA archives, and the original genome annotation. The nucleotide sequences of the putative transposons were retrieved based on their adjacent genes and refining their boundaries according to the sRNA and mRNA reads, which provide insight into their transcriptional activity and possible regulatory elements. To explore the coding potential of these sequences, we performed a six-frame translation. This approach allowed us to capture all potential open reading frames (ORFs) that might encode functional domains/proteins. Subsequently, we determined the conserved protein domains of the coding regions of each TE. Our domain analysis revealed that four of the identified TEs possess hallmark domains of LTR retrotransposons, including protease, reverse transcriptase (RVT), RNase H, and integrase, suggesting that these elements are likely LTR retrotransposons with full coding capacity (Figure 6A and Supplementary Figure S3A). The remaining five TEs displayed only the RVT_1 domain, indicating that they may represent truncated or non-LTR retrotransposons (Figure 6B and Supplementary Figure S3B).

**Figure 7:**
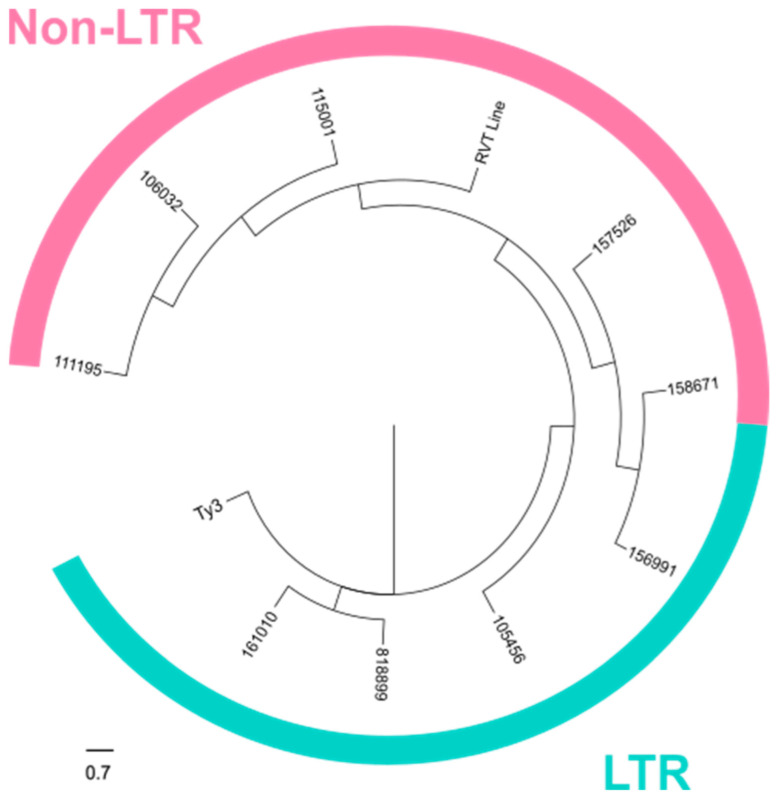
TEs directly regulated by NCRIP. The phylogenetic tree was constructed following the neighbor-joining method. Members of the Ty3/Gypsy and Grem-LINE transposon families were included to establish the phylogeny of the direct NCRIP targets. This included Ty3 (Ty3/Gypsy superfamily) and the RVT Line (*rvt* gene). The LTR transposons are shown in blue while the non-LTR transposons are represented in pink.

To explore the evolutionary relationship between the identified TEs, we conducted a comparative analysis focusing on the reverse transcriptase domain (RVT) (Figure 7). To perform the phylogenetic analysis, we chose a member of each family from the most common transposable elements (LTR and non-LTR) in fungi. From class I, we considered an LTR transposon belonging to the Ty3/Gypsy superfamily (Ty3). Specifically, we used the Ty3/Gypsy polyprotein/retrotransposon from *Rhizoctonia solani* 123E, which is characterized by two long terminal direct repeats (LTRs) flanking the *gag* gene coding a structural protein, and *pol* open reading frame (ORF) coding a polyprotein composed of a protease, a reverse transcriptase and an integrase. Since most of the considered direct targets exhibit zinc-binding in the reverse transcriptase (zf-RVT) domain (PF13966), we also included in this analysis a member of a particular group of genes called Grem-LINEs from *M. lusitanicus* (scaffold_7:3198025-3203971). In the case of these non-LTR TEs, the RVT domain is located within the same open reading frame (ORF) as RNaseH and/or the integrase domains. For the alignment and subsequent phylogenetic analysis, we focused on the domain containing the common RVT. This approach allowed us to directly compare the conserved RVT regions among the TEs, providing a clear understanding of their evolutionary relationships and functions.

We aligned the nine transposons with the control sequences, Ty3/Gypsy and Grem-LINE transposons, using the MUSCLE method, followed by a phylogenetic analysis. As a result, three of the NCRIP targets are phylogenetically similar to the Ty3/Gypsy LTR transposon confirming the structural analysis that we previously performed. Three of the TEs resemble to the Grem-LINE transposon, while the remaining may constitute an LTR-like transposon group, which includes the LTR-transposon ID:156991 (Figure 7). This TE possesses a transposase domain, distinguishing it from the LTR transposons ID:81899, ID:161010, and ID:105456, which are characterized by gag-protease domains instead of a transposase domain (Supplementary Figure S3A).

Since, in other fungi, it has been described that transposons could affect the expression of the neighbor’s genes [25], we analyzed the expression of the genes near these transposable elements to check their silencing effect on the adjacent genes (Figure 6 and Supplementary Figure S3). sRNAs and mRNAs from adjacent genes do not vary between wild-type strain and *r3b2*Δ mutant, so the effect of silencing generated by NCRIP is specific to transposons.

### 2.6. NCRIP Degrades Their mRNA Targets

The claim that NCRIP controls gene expression by direct degradation of specific mRNAs was based on an increase in transcript levels that is associated with a reduction in the transcript-derived sRNA levels in mutants for components of the pathway, such as R3B2, RdRP-1, and RdRP-2 [11]. This idea was reinforced by the ability of R3B2 to bind and cleave single stranded RNA in vitro [16]. However, there was no direct proof that this pathway degrades primary target mRNA in the cell. To confirm this hypothesis, we expressed ectopically polycistronic mRNAs that contained a primary target gene and the firefly luciferase gene, used as a reporter [26], both in the *r3b2*Δ mutant and the wild-type strains. Replacement of the own gene promoter by the heterologous P*zrt1* promoter prevented the experiment readout from being affected by transcriptional regulation. The mRNA expressed by this gene construction contained two independent translational units, the luciferase gene with its own start and stop codons, and the target gene with its own start and stop codons and 3′UTR region, as this region could be involved in regulation or recognition by the NCRIP. Therefore, this mRNA should produce two proteins: the luciferase, the activity of which would be independent of the target protein, and the product of the target gene (Supplementary Figure S4).

For this analysis, we selected three direct targets of NCRIP (ID:81899, ID:112811, and ID:144573) which, in addition to being identified in this transcriptomic analysis, were also detected in a similar independent RNA-seq to identify NCRIP targets at the onset of the phagocytosis by macrophages (our unpublished results), and a gene not controlled by NCRIP as a control (ID:155166). To generate the strains expressing the polycistronic mRNA, the DNA replacement fragment containing the selective marker *leuA* and the polycistronic gene flanked by surrounding sequences of the *carRP* gene was used to transform leucine auxotrophs of the wild-type strain and the *r3b2*Δ mutant (Supplementary Figure S4). Integration in the carotenogenic *carRP* locus produces albino colonies, which are clearly different from the yellow phenotype of both recipient strains [27]. Initial transformants showing albino patches were grown in selective medium for several vegetative cycles to obtain homokaryotic transformants. Two white homokaryotic transformants for each strain were selected and analyzed by PCR, confirming the correct integration of the whole construction in the *carRP* locus (Supplementary Figure S4).

**Figure 8:**
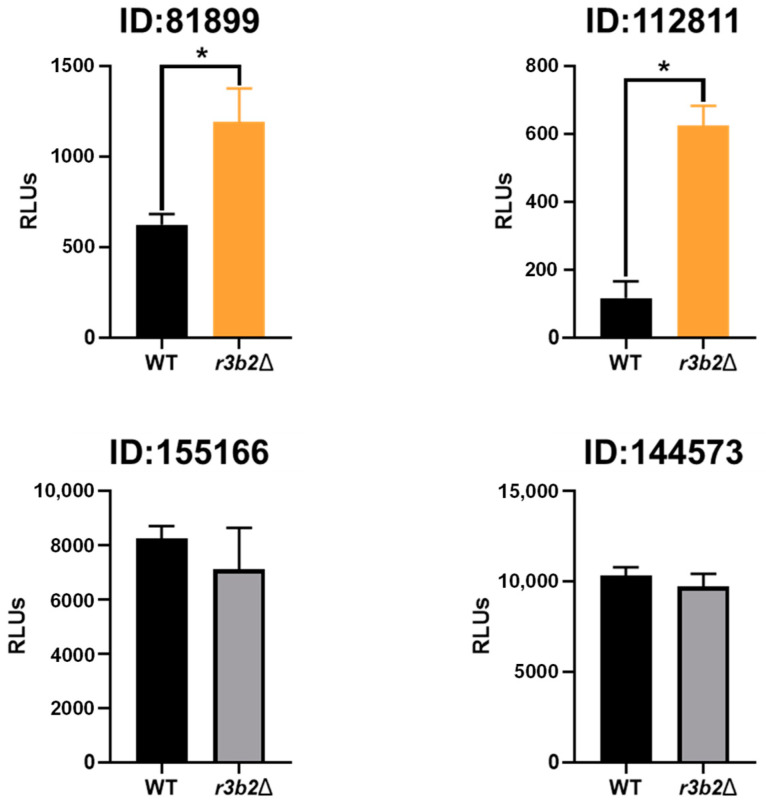
Light emission transformants from the wild-type strain (WT, black bars) and the r3b2Δ mutant (gray bars) expressing polycistronic mRNAs that contained the luciferase gene, and the genes indicated above each bar scheme. ID:81899 (LTR-transposon), ID:112811 encoding an HNH-containing protein, and ID:144573 encoding a putative oxidoreductase were NCRIP direct target, whereas ID:155166 was used as control of NCRIP independent gene. RLUs was determined immediately after adding the luciferase substrate (luciferin) and expressed as mean ± SD. Light emission was measured by FLUOstar. Statistical significance analysis was assessed using an unpaired *t*-test, and the following p-values were obtained: for ID:112811, *p* = 0.0297; for ID:81899, *p* = 0.024; for ID:155166, *p* = 0.3255; and for ID:144573, *p* = 0.0798. The asterisk indicates significant differences (*p* < 0.05).

Light emission as a result of the luciferase activity was higher in the *r3b2*Δ strain than in the wild-type strain for two gene constructions with direct targets of NCRIP (Figure 8), suggesting that these polycistronic mRNAs containing direct NCRIP targets were degraded by this non-canonical RNAi mechanism. Additionally, there was not a null emission in the strains containing the R3B2 protein, revealing that mRNA was not completely degraded.

## 3. Discussion

The NCRIP is an exclusive RNAi pathway of Mucorales that is associated with virulence, making it attractive for new drug design [18]. In this pathway, a unique RNase R3B2 degrades target mRNAs into sRNAs of undefined size [11]. Identification of the direct targets of this RNAi pathway may help to characterize the regulation of NCRIP and to know the signals, or stimuli, involved in its activation or repression. In the present study, we sequenced both sRNAs and mRNAs from the same samples of an *r3b2*Δ mutant and a wild-type strain to identify genes directly regulated by NCRIP under non-stressful conditions, specifically solid rich media for 24 h. We identify nearly one thousand genes regulated by *r3b2* under these conditions, which are mainly involved in metabolic processes, as shown by previous studies [18].

To identify direct targets of the NCRIP, we compared mRNA and sRNA analysis. We define direct targets as genes showing increased mRNA levels and decreased sRNA levels in the *r3b2*Δ mutant compared to the wild-type strain, as the absence of *r3b2* should result in lower targeted mRNA degradation. Under non-stressful conditions, we detected 35 genes that met these criteria, suggesting that they are NCRIP direct targets. The lack of a direct linear correlation between mRNA and sRNAs accumulation suggests that after 24 h, we may be observing the consequences of early NCRIP action. The small number of identified direct targets indicates that most genes regulated by *r3b2* are secondary NCRIP targets or targets of the canonical RNAi pathway as *r3b2* is also required in this RNAi pathway [11]. Half of these DEGs were found differentially expressed in another transcriptomic analysis conducted under entirely different conditions (liquid culture and 48 h of growth), suggesting that they are regulated by this RNAi pathway independently of growth conditions. Again, most of these genes are involved in metabolism, indicating that NCRIP plays an important role in the regulation of metabolism, including the response to oxidative stress [11,18].

The discovery of CAG tandem repeats in the direct targets of R3B2 suggests a potential mechanism for the specific cleavage and regulation of these RNAs within the NCRIP. The CAG repeats may facilitate recognition or binding by R3B2, possibly through the formation of secondary structures such as hairpins, which are known to influence RNA stability and interactions with RNA-binding proteins. Similar to what has been observed in the mutant transcripts of the *huntingtin* (HTT) gene, where expanded CAG repeats fold into hairpin structures. These structures are not stable enough to evade siRNA targeting, leading to efficient silencing of the mutant transcripts compared to normal counterparts [28]. This suggests that the hairpin formations of CAG repeats make them accessible to RNA interference mechanisms, which may be mirrored in the way R3B2 targets its substrates.

For R3B2, which is unique among RNase III enzymes due to its specificity for ssRNAs despite binding to both ssRNAs and dsRNAs [16], the presence of CAG repeats could enhance substrate specificity by promoting the formation of partially double-stranded regions within otherwise single-stranded transcripts. This structural adaptability might not only make the RNAs susceptible to cleavage by R3B2 but also facilitate the generation of esRNAs that are sense to the mRNA, distinct from the canonical RNAi pathways which produce both sense and antisense sRNAs.

In our analysis of the direct NCRIP targets, we identified nine loci containing transposon-related domains. The structural study together with the phylogenetic analysis strongly suggests that these direct targets correspond to transposable elements (TEs) from the LINE-like and LTR transposon families. This observation aligns with the known role of the interference pathways in regulating TE activity, particularly through mechanisms that silence or limit their transposition. The presence of both LINE-like and LTR transposon domains indicates that the NCRIP may target a diverse range of transposon families, which could play a significant role in genome stability and gene regulation under specific conditions. These findings highlight the potential of NCRIP to act as a key regulator not only in controlling transposon proliferation from the LINE family, as was previously described [18], but also in regulating LTR transposons, thus maintaining genomic integrity.

To demonstrate the ability of NCRIP to degrade target mRNAs, we expressed polycistronic mRNAs containing the luciferase gene as a reporter along with different direct NCRIP targets in the *r3b2*Δ mutant and the wild-type strain. Light emission analysis confirmed that mRNAs containing two of three analyzed direct NCRIP targets, including a target with transposable element domains, were degraded in the wild-type strain but not in the *r3b2*Δ mutant. Interestingly, the polycistronic mRNA containing the third direct NCRIP target (ID:144573) did not show significant degradation in the wild-type strain. One possible explanation for this result is that the hybrid mRNA may have acquired a secondary structure that hindered its recognition by NCRIP, preventing its degradation. Alternatively, this target (ID:144573) might require additional sequence elements, RNA-binding factors or specific cellular conditions to be efficiently processed by the pathway. Further analyses, such as structural modeling of the hybrid mRNA or identification of additional cis-regulatory elements, could help clarify the underlying mechanism governing NCRIP specificity. Overall, these experiments suggest that NCRIP is a true degradation pathway that uses RNAi components to regulate the levels of specific mRNAs, mainly those derived from TEs belonging to the LINE and LTR transposon families. Interestingly, direct targets of the NCRIP are characterized by the presence of a motif consisting of CAG tandem repeats that could contribute to the secondary structure formation and contribute to the direct specific mRNAs to be degraded by NCRIP.

## 4. Materials and Methods

### 4.1. Fungal Strains and Cell Cultures

The fungal strains used in this work were derived from *M. lusitanicus* CBS77.49. The wild-type control strain was MU636, and the *r3b2Δ* mutant strain was MU412. These strains are auxotrophic for leucine due to the same mutation in the *leuA* gene and share the same genetic background [11,29]. *M. circinelloides* cultures were grown in rich YPG media at pH 4.5 and 26 °C for optimal growth and sporulation. YNBS 3.2 was used to obtain the transformants [27], and YNB 3.2 was used for luciferase activity assay. The transformants expressing the mRNAs containing the luciferase gene and each of the three direct NCRIP targets and control are indicated in Supplementary Table S1.

The *E. coli* strains were grown in lysogeny broth (LB) medium, supplemented with ampicillin (0.1 mg/mL) if necessary. Solid bacterial cultures were incubated at 37 °C overnight, and the liquid cultures were under the same conditions and at 250 rpm.

### 4.2. RNA-Seq

For RNA extraction, 2.5 × 10^5^ spores were grown in solid YPG at pH 4.5 and 26 °C for 24 h under light conditions. After incubation, three replicates of each sample were pooled to ensure reproducibility. Total RNA, including both sRNA and mRNA, was extracted using the mirVana RNA Isolation Kit (AM1561, Thermo Fisher Scientific, Waltham, MA, USA), following the manufacturer’s procedure. RNA integrity and concentration were assessed through Agilent 2100 Bioanalyzer, (Agilent Technologies, Santa Clara, CA, USA) before proceeding to library preparation.

Library preparation was performed at Novogene using the TruSeq Stranded mRNA HT Sample Prep Kit (Illumina, San Diego, CA, USA), which enriches mRNA via poly(A) selection. The total RNA input was 1 µg per sample, and 8 cycles of PCR were used for library amplification. Libraries were quantified using KAPA Biosystems qPCR Kit (Roche, Basel, Switzerland) and analyzed on a Roche LightCycler 480 real-time PCR instrument (Roche). The libraries were then multiplexed and sequenced using the Illumina NovaSeq 6000 platform (Illumina) with NovaSeq XP v1.5 reagent Kits on an S4 flow cell, following a 2 × 150 bp paired-end sequencing strategy.

For sRNA sequencing, library preparation was conducted at Novogene using Small RNA Sample Prep Kit (Illumina). Unlike mRNA-seq, sRNA molecules do not require fragmentation before library construction. Instead, adapters were ligated directly to the 5′ and 3′ ends of the small RNA sequences. Reverse transcription was then performed to generate a complementary DNA (cDNA) library, followed by PCR amplification to enrich the sRNA sequences. The prepared libraries were multiplexed and sequenced using the Illumina NovaSeq 6000 platform (Illumina), employing a single-end 50 bp (SE50) sequencing strategy to capture short RNA fragments efficiently. The sequencing depth was optimized to achieve a comprehensive representation of the sRNA transcriptome.

### 4.3. Bioinformatic Analyses

#### 4.3.1. sRNA Analysis

The raw sRNAseq reads were quality checked with FASTQC v0.11.8 (http://www.bioinformatics.babraham.ac.uk/projects/fastqc/, accessed on 17 September 2024) before and after removing adapter (3’) and contaminant sequences. Low-quality reads (Phred quality Q < 32) and reads shorter than 13 nucleotides were removed using TrimGalore! v.0.4.3.1 (https://github.com/FelixKrueger/TrimGalore, accessed on 17 September 2024).

Following preprocessing, clean reads were aligned to the *M. circinelloides f. lucitanicus* v2.0 genome using Bowtie2 v2.5.3 [30] The reference genome was indexed using Bowtie2 prior to alignment to facilitate efficient and accurate read mapping.

For the quantification of reads mapped to *Mucor* genes, FeatureCounts v2.0.1 [31] tool was used. The software produced a count table file (with several reads from each library that defined each locus). This count table served as input for the differential expression (DE) analysis between the mutant and wild-type strains using DESeq2 v2.11.40.8 tool [32]. We considered as differentially expressed the loci with a false discovery rate (FDR) adjusted *p*-value ≤ 0.05 and log2 fold change [log_2_FC] >= |1.0|.

#### 4.3.2. mRNA Analysis

Raw mRNA datasets were checked for quality with FASTQC before and after removing adapter and contaminant sequences with TrimGalore! v.0.4.3.1 excluding reads with a Phred quality score Q ≤ 32 and/or a total length ≤ 20 nucleotides as well as adapter sequences with an overlap ≥ 4 bases. The mRNA clean reads were aligned to the *M. circinelloides f. lucitanicus* v2.0 genome using HISAT2 aligner [33], which uses a graph-based alignment approach to achieve high sensitivity and accuracy. HISAT2 employs both a global GFM index and a large set of smaller, local indexes that cover the whole genome, allowing for efficient alignment, especially for reads spanning multiple exons. Gene expression levels were quantified by counting the number of mapped reads to specific genes. However, read count alone is not sufficient to represent gene expression levels as it is influenced by factors such as gene length and sequencing depth. Therefore, the FPKM (fragments per kilobase of transcript per million mapped reads) metric was used to normalize for these factors, making gene expression estimates comparable across different genes and experiments.

To further analyze gene expression, the HTSeq software v2.0.5 [34] was used in union mode to calculate read counts, and FPKM values were derived for each gene. For differential gene expression analysis, several steps were carried out. Read count normalization: normalization was applied to adjust for differences in sequencing depth and other technical factors that could skew expression measurements. Model-dependent *p*-value estimation: differential expression between conditions was tested using a statistical model, and *p*-values were calculated to determine the significance of gene expression changes. False discovery rate (FDR) estimation: to account for multiple hypothesis testing, the FDR was calculated, and genes with an FDR ≤ 0.05 were considered significantly differentially expressed. Additionally, genes with a log2 fold change (log_2_FC) ≥ |1.0| were considered to exhibit significant expression changes.

Transposons and their adjacent gene expression analysis were performed using bamCoverage tool v3.3.2.0.0 to normalize coverage to bins per million reads (BPM) in 25 bp bins, thus, dividing the genome into 25 bp bins. The bigWig files produced by bamCoverage were visualized using pyGenomeTracks (v3.6).

#### 4.3.3. Functional Analysis

KOG class enrichment analyses were performed in Prism; delta-ranks were computed as the difference between the mean of all genes within the KOG class and the mean rank of all other genes in a Mann–Whitney U-test. A KOG class was considered over-represented if *p* ≤ 0.05 in one-sided Fisher’s exact evaluation of the DEGs compared to the total number of genes in each KOG class.

GO terms related to the molecular biology (MB) and biological processes (BP) of the direct targets of the NCRIP were obtained from the GO annotation. To visualize the relationships and GO terms of these genes, a network was constructed using Cytoscape v3.10.3 [35], incorporating expression data from the transcriptomic analysis of sRNA and mRNAs.

#### 4.3.4. Prediction of RNA Motifs in the Direct Targets of the NCRIP

DNA sequences of the direct targets were retrieved using bedtools getfasta tool. Subsequently, DNA was converted to RNA. The RNA sequences were then analyzed for conserved motifs using the MEME suite [36].

#### 4.3.5. Phylogenetic Analysis

The reverse transcriptase (RVT) domains found in the transposable elements were aligned with MUSCLE [37]. A neighbor-joining tree was inferred from this alignment using the JTT substitution model and a bootstrap procedure of 1000 iterations (MEGA v 10.0.5) [38].

### 4.4. RT-qPCR Quantification

For RT-qPCR, about 5 µg of total RNA of wild-type and mutant strain samples were treated with Turbo DNase (Thermo Fisher). The RNA samples were routinely checked for DNA contamination by a PCR analysis using primers for the housekeeping elongation factor gene. For cDNA synthesis, 1 µg of total RNA was reverse-transcribed using the iScript cDNA synthesis kit (Bio-Rad, Hercules, CA, USA) at 25 °C for 10 min, 42 °C for 50 min, and 70 °C for 15 min. The RT-qPCR was performed in triplicate using 5X SYBER green PCR master mix (Applied Biosystems, Thermo Fisher Scientific Inc., Waltham, MA, USA) with a QuantStudio TM 5 flex system (Applied Biosystems) following the supplier’s recommendations. To ensure non-specific amplification, non-template control and melting curve were tested. The primer sequences used for the quantification of genes are listed (in Supplementary Table S2). The efficiencies of every pair of primers were approximately identical; the relative expression of target genes was obtained by the delta–delta cycle threshold (ΔΔCt) method, normalizing for the endogenous control elongation factor (*ef*).

### 4.5. Plasmid Constructions

The plasmid pMAT1904 carries the luciferase gene under the control of the strong promotor *zrt1* gene (P*zrt1*) from *M. lusitanicus* and the selection marker *leuA*. Selected genes were cloned using primers with restriction sites for the enzyme SacII for subsequent gene digestion and cloning in pMAT1904. On both sides of the luciferase gene and the each NCRIP target, there are *carRP* gene sequences to facilitate the integration of the whole construct in this *M. lusitanicus* locus. The plasmids obtained are in Supplementary Table S3.

### 4.6. Mucor Transformation

In this work, two recipient strains were used: MU636 (*leuA*^−^) and MU412 (leuA^−^, *r3b2*Δ). Transformants were obtained following the previous transformation procedure [27]. In short, harvested fresh spores were incubated for 2–4 h, ensuring their correct germination. After, protoplasts were achieved by cell wall digestion of the germinated spores with lysing enzymes (Merck, Darmstadt, Germany) and chitosanase (Merck). Protoplast transformation was performed by electroporation with SalI linearized plasmids and later incubated in poor agar medium YNBS 3.2 and checked after 3–4 days for colonies. The right construct integration disrupts the *carRP* gene, involved in carotenoid biosynthesis, producing albino colonies. Those white colonies were severally transferred to fresh YNB agar plates (5–10 cycles) to obtain homokaryons.

### 4.7. Transformants Screening by PCR

DNA from transformants was extracted as previously described. Integration of the gene constructions was checked by PCR using primers that aligned to the luciferase gene and the *carRP*.

### 4.8. Luciferase Assay

Luciferase activity was tested in vitro following the procedure developed by Urlike et al. [26]. Light emission was measured by FLUOstar Omega luminometer (Thermo Fisher). Bioluminescent strains were cultured in YNB 3.2 in a total of 100 µL at 2 × 10^5^ spores/mL for 24 h in 96-well microtiter plates (Nunc, Thermo Fisher), at 26 °C and under light, 50 µL of luciferin (Synchem, D-luciferin, synthetic luciferin) were added to the cultures and light emission was immediately measured, before the measuring plate was shaken orbitally at 100 rpm.

To normalize light emission against fungal biomass, we used a 3.5% (vol/vol) calcofluor-white stain (18909-100ML-F, Sigma Aldrich, Burlington, MA, USA) and measured fluorescence using the Omega luminometer with excitation and emission lengths of 360 nm and 485 nm, respectively.

## Figures and Tables

**Table 1:** Gene groups regulated by *r3b2*.

	sRNA Increased	sRNA Decreased	No Match with sRNA	Total mRNA
mRNA upregulated	239	35	373	647
mRNA downregulated	0	173	169	342
No match with mRNA	189	962		
Total sRNA	428	1170		

## Data Availability

The data that support the findings will be available through NCBI once the manuscript is accepted for publication. All datasets generated in this study are available at our GitHub page https://github.com/ghizlanetahiri95/NCRIP_targets (accessed on 13 February 2025).

## Supplementary Materials

The following supporting information can be downloaded at: https://www.mdpi.com/article/10.3390/ijms26062738/s1.
