# Supplementary material for: Direct Targeted Degradation of Transposon RNAs by the Non-Canonical RNAi Pathway of the Fungus Mucor lusitanicus
Source: Int J Mol Sci. 2025 Mar 18;26(6):2738. doi: 10.3390/ijms26062738 (PMC11943222; doi:10.3390/ijms26062738)
Supplement: Supplementary file 1 [file ijms-26-02738-s001.zip › Supplementary Material.docx]

Supplementary material for

Direct targeted degradation of transposon RNA by the non-canonical RNAi pathway of the fungus *Mucor lusitanicus*


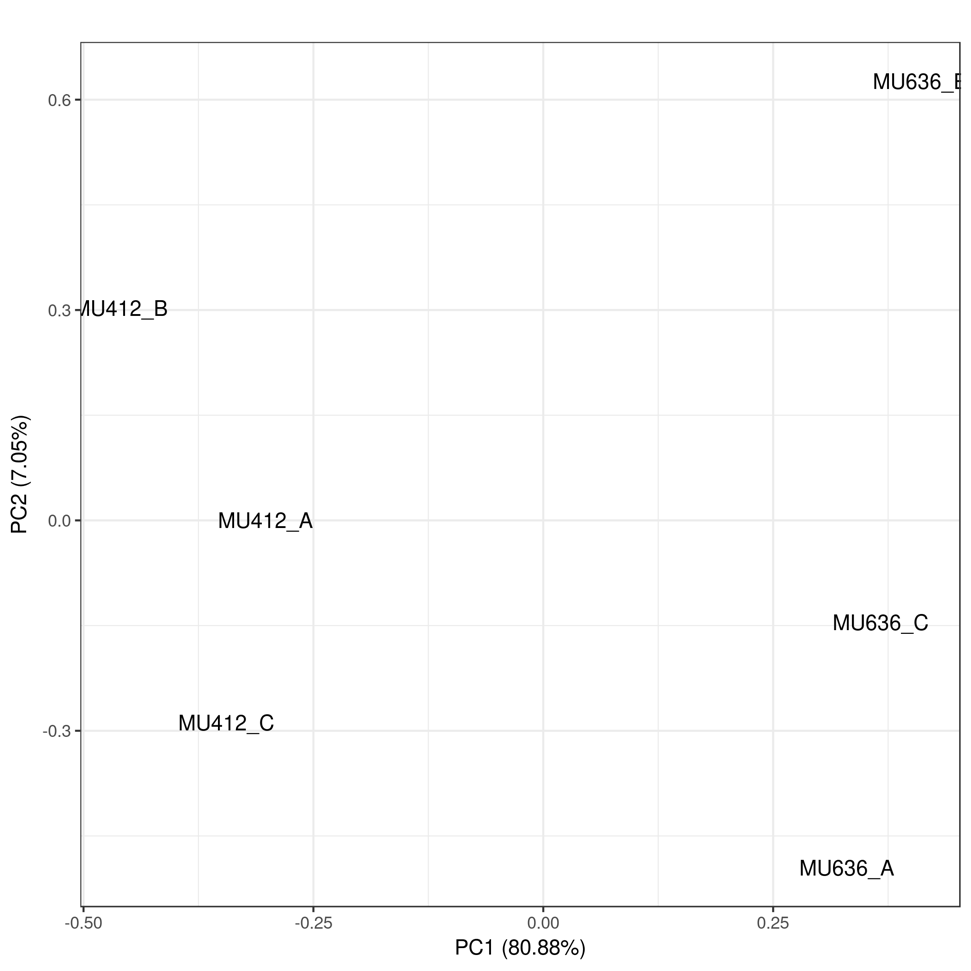


MU636_B

MU412_B

**Figure S1**. Principal Component Analysis (PCA) to determine the similarities between RNA-seq samples of the *r3b2Δ* (MU412) mutant and the wild-type strain (MU636).


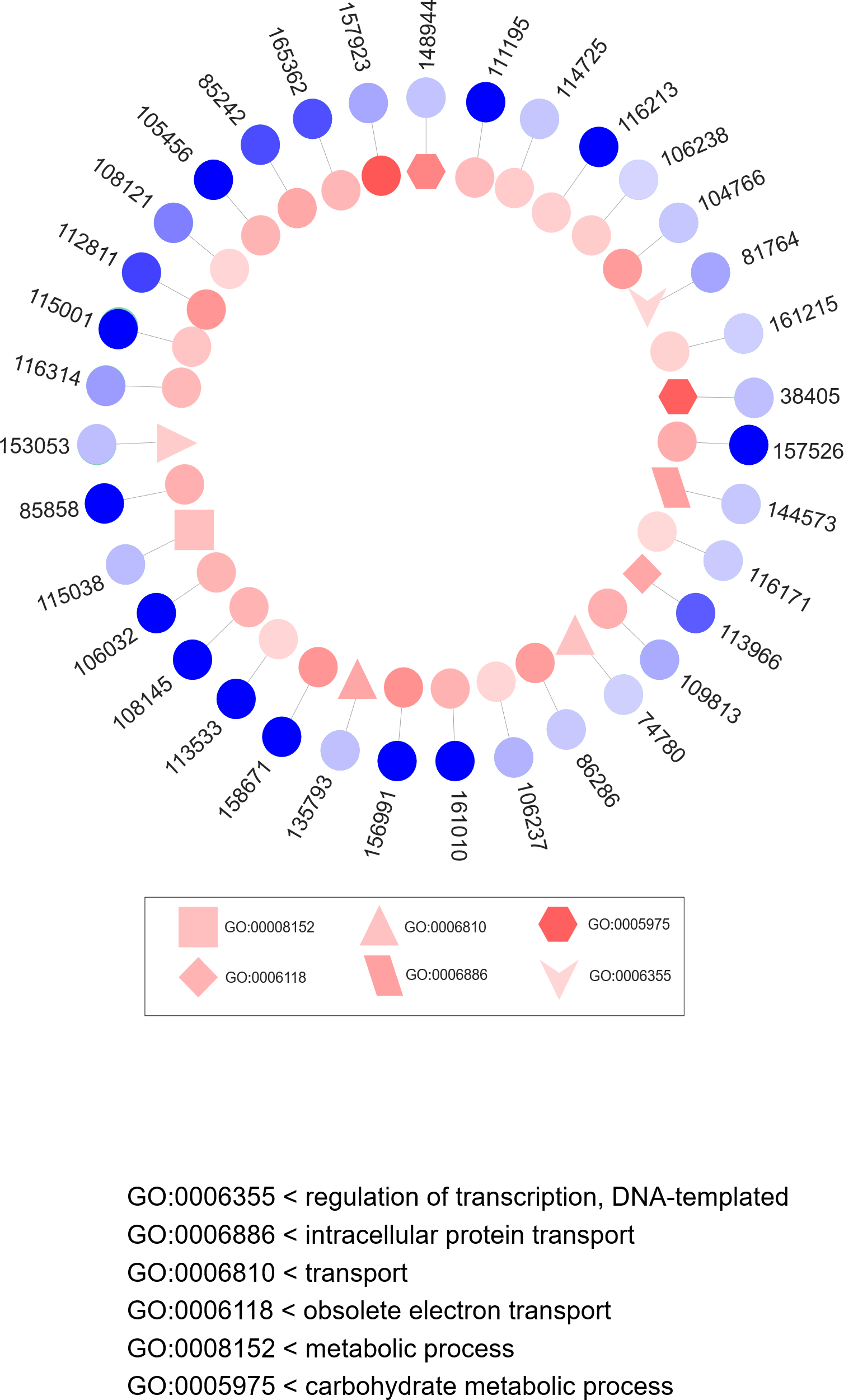


**Figure S2**: Biological Processes GO terms associated to the NCRIP targets. The internal layer of the plot represents the expression levels of these genes at the mRNA level, while the external layer shows the expression at the sRNA level. Circular shapes within the internal layer indicate genes with unknown molecular functions, whereas other shapes correspond to genes with identified molecular functions.


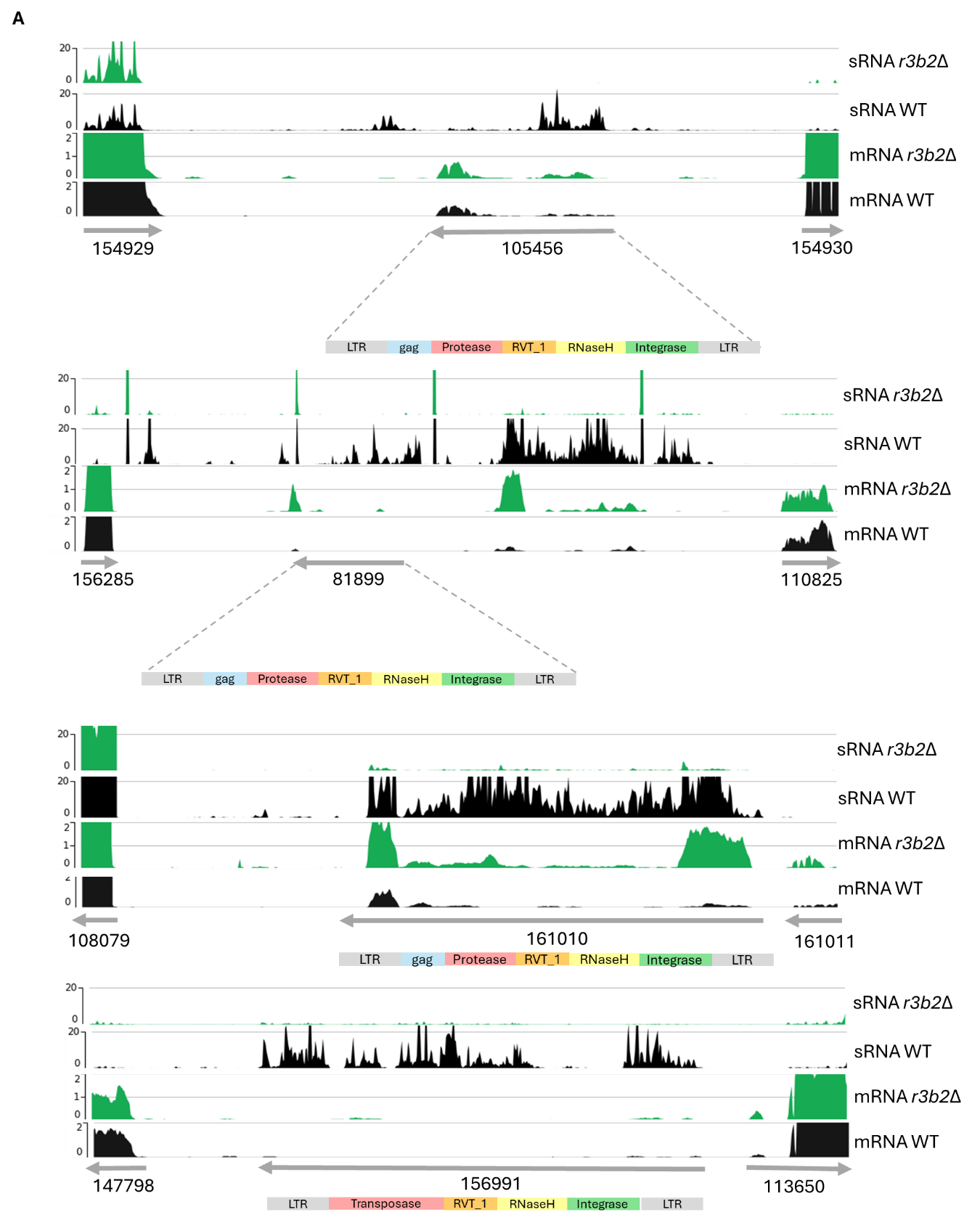


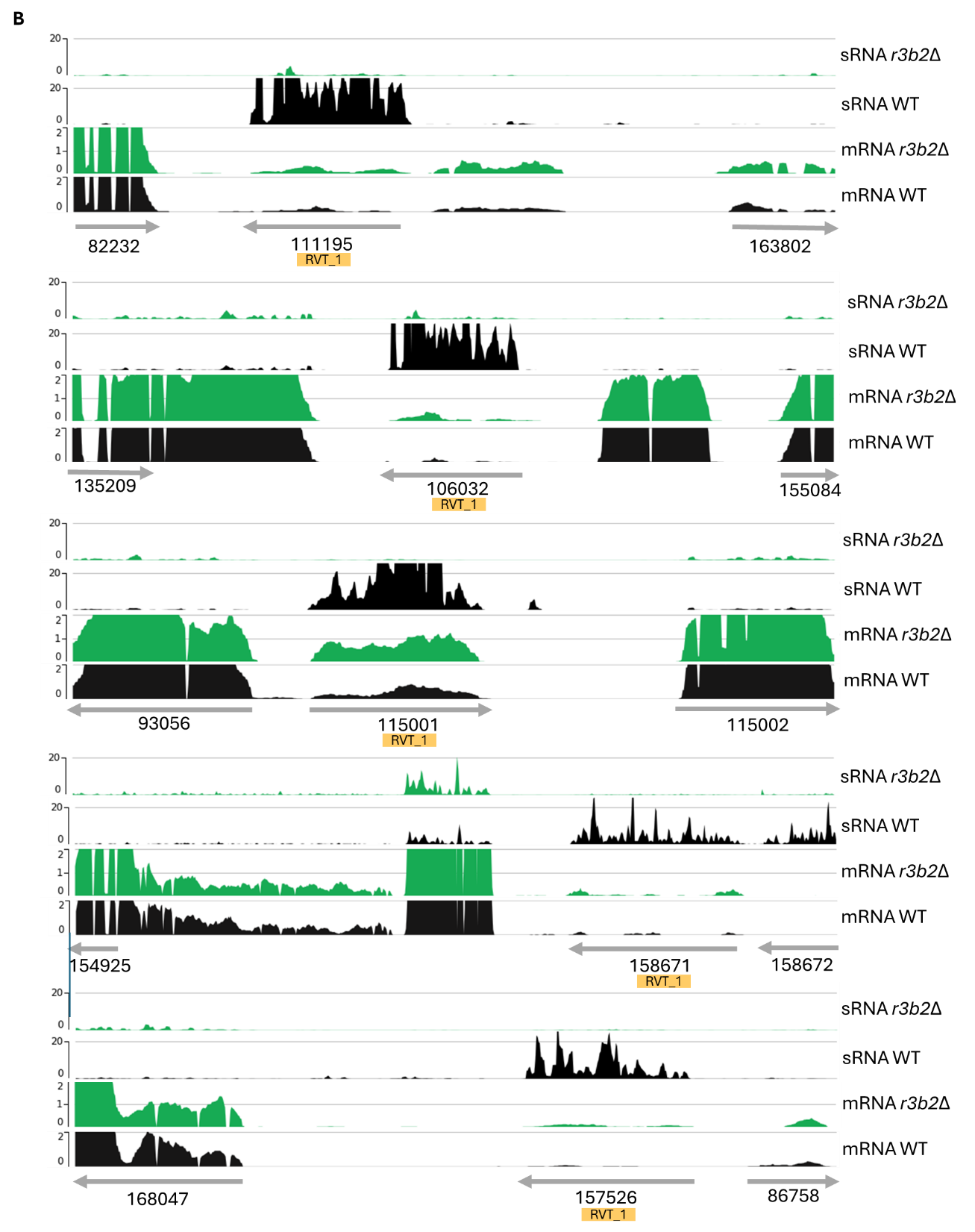


**Figure S3.** Annotation of transposable elements directly targeted by the NCRIP. **(A)** Structure of putative LTR transposon showing its key structural domains, including the possible Long Terminal Repeats (LTRs), and the conserved protein domains such as protease, reverse transcriptase (RVT_1), RNase H, and integrase. Green and black plots show the coverage of sRNA and mRNA reads mapped to the transposon and their adjacent genes in the WT and mutant (∆r3b2) samples, respectively. **(B)** Structure and expression of non-LTR transposon. In both cases the same data from the neighboring genes are shown.


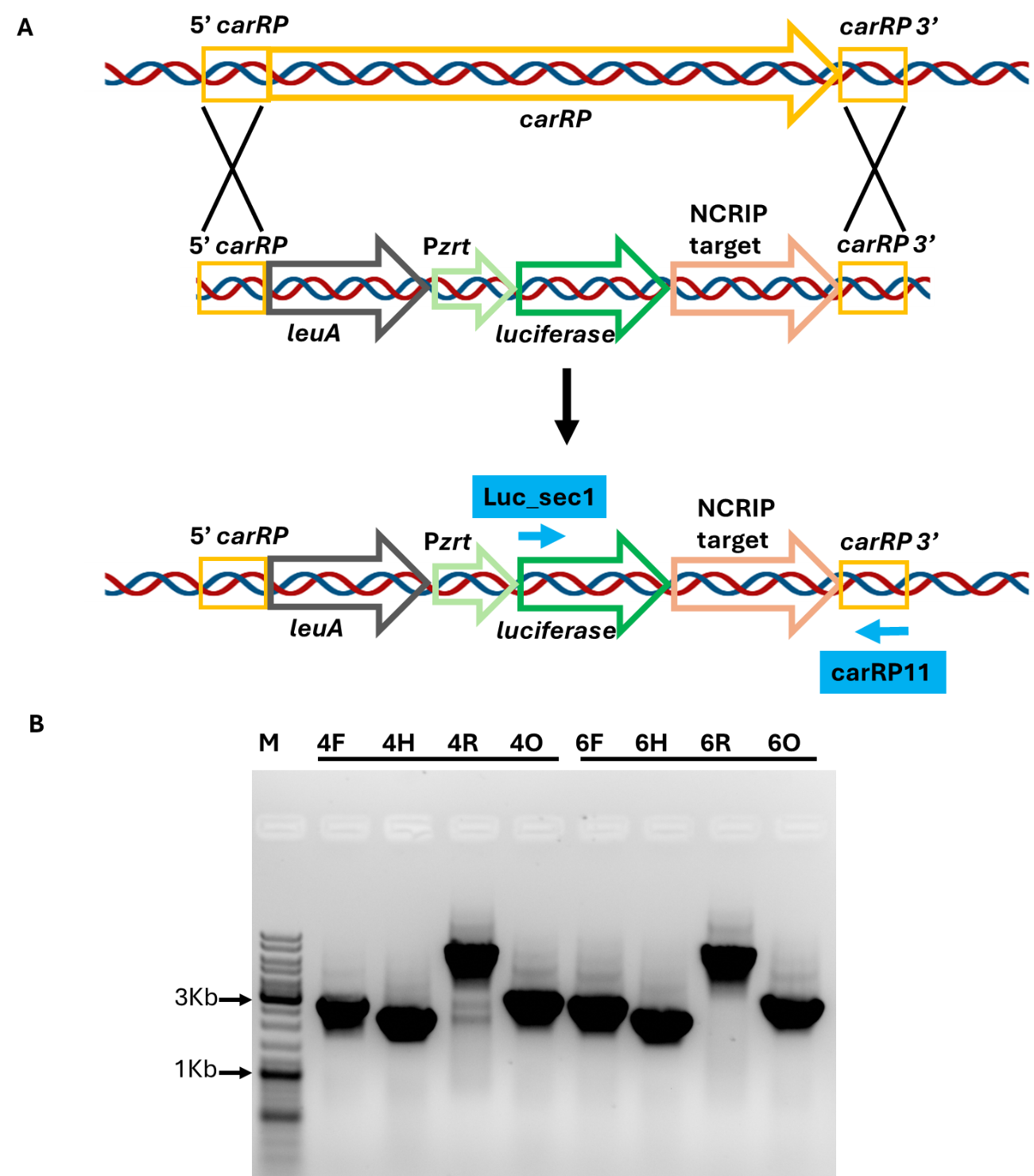


**Figure S4**: **(A)** Genetic construction of the polycistronic mRNAs containing a direct target of the NCRIP and the *luciferase* gene reporter. **(B) PCR verification, with primers Luc_sec1 and carRP11 of the t**ransformants 4F, 4H, 4R, and 4O producing polycistronic mRNA containing ID:144573, ID:112811, ID:81899, and ID:155166, respectively, in the r3b2∆ strain. The same analysis for transformants 6F, 6H, 6R, and 6O producing the same polycistronic mRNA in the wild-type (WT) strain.

**Table S1**. Transformants obtained in this work.

| Recipient strain | Plasmid | Gene ID |
| --- | --- | --- |
| *r3b2Δ* | pMAT1905 | 144573 |
| *r3b2Δ* | pMAT1906 | 155166 |
| *r3b2Δ* | pMAT1907 | 112811 |
| *r3b2Δ* | pMAT1908 | 81899 |
| wild-type | pMAT1905 | 144573 |
| wild-type | pMAT1906 | 155166 |
| wild-type | pMAT1907 | 112811 |
| wild-type | pMAT1908 | 81899 |

**Table S2**. Primers used in this work.

| ID | Forward | Reverse |
| --- | --- | --- |
| 114483 | GlutathioneFw  CCTTCATCTTTGGTGACGAG | GlutathioneRv  CGTAGGTCTGCTTTGAATAGC |
| 152804 | VesicleFw  GCTCGTGCTAAGCGTATGAGAG | VesicleRv  CTTGGACCAGCTCAGAGACG |
| 156292 | RVTFw  GCTCTAATCAACTGGCTATGGC | RVTRv  ATGAGCAGGTGAGGAGCATC |
| 81899 | Retro_Fw_Sac2 tATCTCCGCGGCTCTGGTCAACGAGACATTGC | Retro_Rv_Sac2  tacgctccgcggGTTGTTGAGTTATATGGCTCTG |
| 115166 | Oxy_Fw_Sac2  AgctaccgcggATGGTAAGACAGCAGATCAGAG | Oxy_Rv_Sac2  ACTGACCGCGGGATTGCCACACTGCCATCAAAGC |
| 112811 | HNH_Fw_Sac2  tcgtaccgcggATTCAATGAACAATCGTGCTTC | HNH_Rv_Sac2  gtactaccgcggGTGTTACCAGTGCACAGACGATG |
| 144573 | FLAV_Fw_Sac2  taagaccgcggAATGTCTATTACTACTGCCACtac | FLAV_Rv_Sac2  cgtgaccgcggGTATTTAAATTAGAAACAGAG |

**Table S3.** Plasmids obtained in this work.

| Name | Containing gene (ID) |
| --- | --- |
| pMAT1905 | 144573 |
| pMAT1906 | 155166 |
| pMAT1907 | 112811 |
| pMAT1908 | 81899 |
